# Temporal Patterns of High-Spend Subgroups Can Inform Service Strategy for Medicare Advantage Enrollees

**DOI:** 10.1007/s11606-021-06912-4

**Published:** 2021-06-07

**Authors:** Samuel J. Amodeo, Henrik F. Kowalkowski, Halley L. Brantley, Nicholas W. Jones, Lauren R. Bangerter, David J. Cook

**Affiliations:** 1 OptumLabs at UnitedHealth Group, Minneapolis, MN USA; 2 Department of Health Services, Policy, and Practice, Brown University, Providence, RI USA

**Keywords:** Medicare Advantage, high-cost patients, segmenting, complex patients

## Abstract

**Background:**

Most healthcare costs are concentrated in a small proportion of individuals with complex social, medical, behavioral, and clinical needs that are poorly met by a fee-for-service healthcare system. Efforts to reduce cost in the top decile have shown limited effectiveness. Understanding patient subgroups within the top decile is a first step toward designing more effective and targeted interventions.

**Objective:**

Segment the top decile based on spending and clinical characteristics and examine the temporal movement of individuals in and out of the top decile.

**Design:**

Retrospective claims data analysis.

**Participants:**

UnitedHealthcare Medicare Advantage (MA) enrollees (*N* = 1,504,091) continuously enrolled from 2016 to 2019.

**Main Measures:**

Medical (physician, inpatient, outpatient) and pharmacy claims for services submitted for third-party reimbursement under Medicare Advantage, available as International Classification of Diseases, Tenth Revision, Clinical Modification (ICD-10-CM) and National Drug Codes (NDC) claims.

**Key Results:**

The top decile was segmented into three distinct subgroups characterized by different drivers of cost: (1) *Catastrophic*: acute events (acute myocardial infarction and hip/pelvic fracture), (2) *persistent*: medications, and (3) *semi-persistent* chronic conditions and frailty indicators. These groups show different patterns of spending across time. Each year, 79% of the catastrophic group dropped out of the top decile. In contrast, 68–70% of the persistent group and 36–37% of the semi-persistent group remained in the top decile year over year. These groups also show different 1-year mortality rates, which are highest among semi-persistent members at 17.5–18.5%, compared to 12% and 13–14% for catastrophic and persistent members, respectively.

**Conclusions:**

The top decile consists of subgroups with different needs and spending patterns. Interventions to reduce utilization and expenditures may show more effectiveness if they account for the different characteristics and care needs of these subgroups.

**Supplementary Information:**

The online version contains supplementary material available at 10.1007/s11606-021-06912-4.

## INTRODUCTION

Most healthcare costs are concentrated among a small proportion of individuals. The most expensive (top decile) group of enrollees account for about 68% of total healthcare costs (range: 55–77%).^1^ Many interventions that target the top decile have shown limited effectiveness in reducing healthcare utilization and spending. The Camden Coalition program sought to reduce spending and improve healthcare quality among frequent healthcare utilizers through interdisciplinary care coordination of outpatient care but proved ineffective in reducing 180-day readmissions.^2^ The SafeMed program approached the same aims through early identification and patient engagement in the hospital followed by intensive community-based follow-up post-discharge. SafeMed Medicaid enrollees experienced decreases in emergency department (ED) visits, hospitalizations, and 30-day readmissions, but this was not observed for MA enrollees.^3^ High-cost individuals may be targeted by payer-based programs that offer increased care and case management based on their high-cost status. However, targeting individuals based on cost alone does not account for patient characteristics and is likely to result in wasted resources, diminished returns, potentially misdirected interventions, and poorer health outcomes.

One reason that interventions have seen inconsistent efficacy is due to temporal changes in cost. Among high-spend individuals who overuse emergency departments and hospital services, high utilization often lasts less than a year, regardless of intervention.^4^ Another reason that interventions fail to reduce spend is the substantial variation in potentially preventable spending within the high-spend population.^5–8^ Khullar et al. found that preventable spending among high-cost individuals is concentrated on patients who were seriously ill, frail, or had a serious mental illness.^8^ Preventable cost also varies significantly by care setting.^9^

Segmenting the top decile provides an opportunity to identify subgroups who would benefit most from care, case, or disease management, or palliative care.^10–12^ Previous research has divided high-spend, high-need individuals into meaningful cost groups.^13–16^ Powers et al. identified ten high-cost subgroups based on conditions.^14^ Each group had different patterns of utilization, spending, and mortality, suggesting that better segmentation could inform strategies to decrease spending.^14^ Hayes et al. segmented individuals into groups based on chronic diseases and functional limitations and found that higher needs (defined as three or more chronic diseases and functional limitations) were linked to greater healthcare spending and out-of-pocket costs.^17^ Previous studies have focused on segmenting individuals using a single time window;^8,14,15,18^ less work has focused on the multi-year temporal persistence of high-cost individuals. The present study segments the top cost decile of MA enrollees based on spending patterns and clinical criteria derived from existing literature. We examined characteristics of individuals in these groups and the temporal movement of individuals in and out of the highest cost decile using de-identified claims data from 2016 to 2019. This approach draws from Pearl and Madvig, who identified three groups whose needs can be proxied by their spending patterns^19^:

1. Patients with chronic conditions whose spending fluctuates
2. Chronically ill patients who require expensive, ongoing treatment
3. Healthy patients who experience a catastrophic medical event

## METHODS

### Data

Analyses were conducted using aggregated de-identified administrative claims from 2016 to 2019 for MA-insured individuals in a research database from a single large US health insurance provider (the UnitedHealth Group Clinical Discovery Portal). The database contains medical (physician, inpatient, outpatient) and pharmacy claims for services submitted for third-party reimbursement from Medicare (Medicaid claims not included if dually enrolled). Because no protected health information was extracted or accessed during the study and all data were accessed in compliance with the Health Insurance Portability and Accountability Act, institutional review board approval or waiver of authorization was not required. (See Appendix in the Supplementary information for additional details on database quality.)

**Figure 1:**
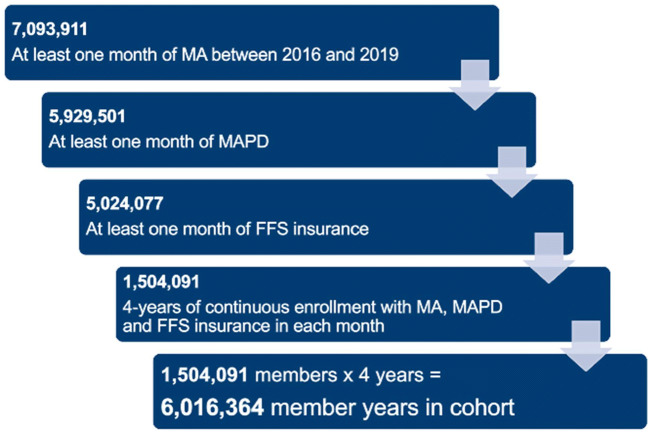
Waterfall representing cohort selection criteria. Acronyms: MA, Medicare Advantage; MAPD, Medicare Advantage Part D; FFS, fee-for-service

The population was restricted to individuals continuously enrolled (enrollment does not have gaps of a month or longer) in a non-capitated Medicare Advantage Part D (MAPD) plan. The population also excluded all previous and current UnitedHealth Group employees. Continuous enrollment excluded the 3–5% of enrollees who died each year between 2016 and 2019. This resulted in a sample size of 1,504,091 (Fig. 1). We then performed an ad hoc analysis of mortality rates within subgroups that was inclusive of deaths and required continuous enrollment until the month of death, resulting in a sample of 1,701,647 individuals.

Data across six dimensions were examined: cost, demographics, service locations, medications, utilization, and diagnoses. Cost features included total healthcare cost, spending on outpatient and inpatient care, physician visits, and medication. Race was obtained from the Centers for Medicare and Medicaid Services (CMS) Monthly Membership Report. Socioeconomic status^20^ and rural, urban, or suburban status was obtained using zip code.

Thirty-three chronic conditions were flagged using ICD-10 diagnosis codes derived from the CMS Chronic Conditions warehouse.^21^ We used the claims-based frailty indicators detailed and coded in Gilbert et al.,^22^ which include seven components of frailty (falls and fractures, anxiety and depression, mobility problems, dementia and delirium, pressure ulcers and weight loss, dependence and care, and incontinence). CPT and HCPCS procedure codes were aggregated into 244 clinically meaningful categories using the Clinical Classification Software developed by the Agency for Healthcare Research and Quality (AHRQ) Healthcare Cost and Utilization Project (HCUP).^23^ Medications identified through pharmacy claims were aggregated using the first two digits of the American Hospital Formulary Service (AHFS) Pharmacologic-Therapeutic Classification code.^24^

### Analysis

Cost thresholds were determined by deciles of total individual healthcare costs each year. The thresholds were used to separate the population into ten equally sized cost groups, used to examine transitions between deciles across time. We identified clinical and utilization characteristics to segment individuals based on their likelihood of remaining in the top decile year to year. Through exploratory analysis, literature review, and consultation with clinicians, we segmented the individuals in the most expensive decile into three groups:

1. Catastrophe: Individuals were labeled catastrophic in year *t* if their annual cost was below the 60th percentile in year *t*−1 and at or above the 90th percentile in year *t.* The 60th percentile was chosen as the lower bound threshold because it resulted in a 40% or greater increase in mean and median cost from 1 year to the next and captured over 20% of individuals in the top decile. The criteria for this group were informed by the discussion of catastrophic individuals by Pearl and Madvig.^25^
2. Persistent: Individuals were labeled persistent if they were not already classified as catastrophic in year *t*, had spent at or above the 90th percentile in year *t*, and met one of the following criteria:
  - Had dialysis or end-stage renal disease (ESRD). It is well documented that individuals with ESRD are persistently high-spend.^26^
  - Used a drug from the list of AHFS chronic drug categories below. Usage of such drugs suggests ongoing, expensive, chronic disease management for individuals that is unlikely to change.^27^
    i Antineoplastic agents
    ii Disease-modifying antirheumatic agents (DMARDs)
    iii Phosphate-removing agents
    iv Immunomodulatory
    v HIV nucleoside/nucleotide reverse transcriptase inhibitors
  - Had pharmacy cost that was at or above 60% of their total healthcare cost for the year. High pharmacy cost ratios have been demonstrated to be strongly predictive of high overall healthcare costs.^14^
  - Were < age 65 in 2016. Medicare covers people under 65 who receive Social Security Disability Insurance or are diagnosed with ESRD or amyotrophic lateral sclerosis (ALS). This population has shown persistently high cost.^15^
3. Semi-persistent: Individuals were semi-persistent if they were not catastrophic or persistent.

We examined the temporal persistence in each group across 3 years (2017–2019). The groups could not be identified in 2016 due to catastrophic criteria requiring 2 years of data. We compared demographics, geographic location, comorbidities, and healthcare utilization across the groups. We analyzed pharmacy spend and frailty as two key drivers of cost. Characteristics and utilization were analyzed for 2017–2019; 2017 was representative of the other years and was chosen to illustrate the defining features of the groups. We conducted chi-square tests of independence for the categorical variables and analysis of variance (ANOVA) tests for the continuous variables to determine significance of each comparison.

We use multinomial logistic regressions on data from 2016 to 2018 to compute odds ratios for the most impactful characteristics of those within each spend group in the next year. To verify the criteria for the persistent group, we use multinomial logistic regressions on data from 2016 to compute odds ratios for the most impactful characteristics of those with 3 years in the top decile of spend from 2017 to 2019. The specifics of validating the tests and more details on the two-step process of variable selection and odds ratio evaluation for each model are included in the Appendix in the Supplementary information. Analyses were conducted using Python and the statistical software R.^28^

## RESULTS

**Figure 2:**
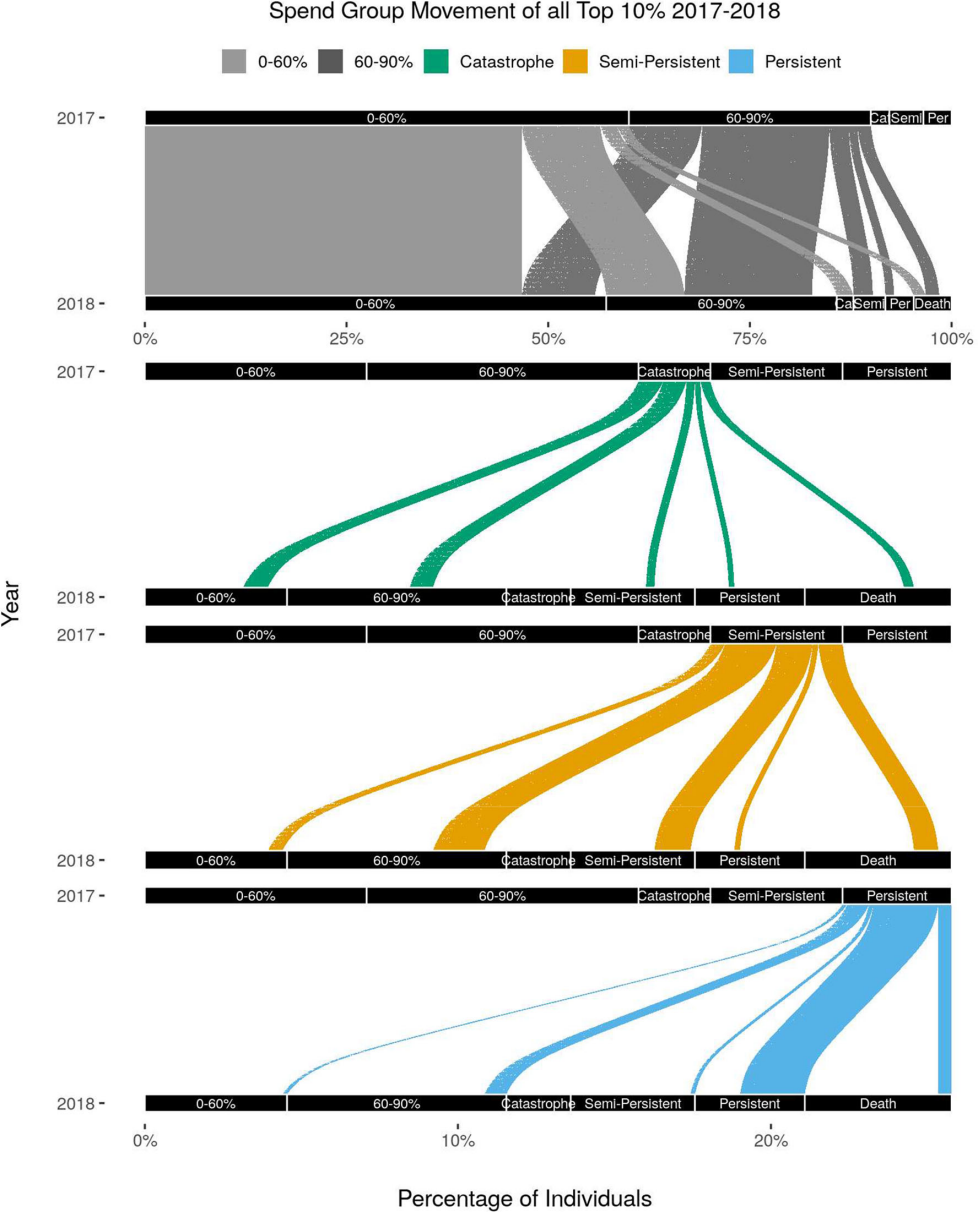
Movement of individuals in and out of spend categories in 2017–2018. Trends are representative of 2018–2019 as well. Notes: All individuals are represented in the top panel. The bottom three panels only represent members that moved into or out of the top decile or died in 2018. (In the top panel, Ca is catastrophe, Semi is semi-persistent, and Per is persistent).

The cohort skewed females (58.3%) and had an average age of 73.0 years (S.E. 0.01) in 2017. Most of our sample identified as White (79.6%) followed by Black (13.4%), Other (1.9%), Asian (1.7%), Hispanic (1.6%), and Native American (0.2%). The sample was distributed across suburban (39.3%), rural (32.3%), and urban (28.4%) regions. Analysis of cost transitions found that individuals were more likely to stay in the same decile. Individuals in the lowest and highest deciles had the most stability year over year. This trend was consistent across time: from 2016 to 17, 2017–18, and 2018–19, the proportion of individuals that remained in the top decile from 1 year to the next was 43%, 45%, and 45% respectively (Fig. 2, Appendix B in the Supplementary information). Individuals who did change decile from year to year were likely to move to an adjacent decile (dropping from 90–100% to 80–90%).

Demographic and healthcare utilization characteristics are shown in Table 1. The persistent group contains the greatest proportion of individuals under 65 (46.9%) as well as individuals who are dual-eligible (eligible for both Medicare and Medicaid; 25.99%). Compared to the other groups, the persistent group contained more individuals that identified as Black (22.8%) and a smaller proportion of individuals that identified as White (71.6%) (*p*-values <0.001). Geographic region (rural, suburban, urban) did not differ materially between the three groups.

**Table 1:** Demographics and Healthcare Utilization of Individuals in the Lower Nine Spend Deciles and Catastrophic, Persistent, Semi-Persistent Spend Groups. Values Are Shown for 2017, But Trends Are Representative of All Years (2017–2019)

Characteristics	Bottom 90%	Top 10%		
		Catastrophic	Persistent	Semi-persistent
	*n* = 1,353,681	*n* = 33,534	*n* = 53,749	*n* = 63,127
Female	58.38%	52.94%	58.98%	58.87%
Age, *M* (S.E.)	73.11 (0.01)	74.56 (0.05)	65.14 (0.05)	76.49 (0.03)
<65	9.72%	8.62%	46.91%	0.00%^d^
65–75	54.98%	48.51%	35.87%	50.56%
>75	35.29%	42.87%	17.22%	49.44%
SES, *M* (S.E.)^a^	52.52 (<0.01)	52.57 (0.02)	52.25 (0.02)	52.50 (0.02)
Dual-eligible	8.36%	9.41%	25.99%	13.59%
Race				
Asian	1.77%	1.16%	0.93%	0.85%
Black	13.02%	12.45%	22.78%	12.85%
Hispanic	1.61%	2.29%	1.84%	0.86%
Native American	0.12%	0.14%	0.25%	0.20%
Other	1.97%	1.66%	1.28%	1.26%
White	79.69%	80.70%	71.59%	82.78%
Missing	1.80%	1.60%	1.34%	1.20%
Geographic region				
Rural	32.04%	29.47%	37.86%	34.37%
Suburban	39.41%	37.48%	36.91%	39.32%
Urban	28.51%	33.01%	25.20%	26.26%
Missing	0.04%	0.03%	0.03%	0.05%
Healthcare utilization, *M* (S.E.)				
Number of frailty indicators	0.46 (<0.01)	1.26 (0.01)	1.08 (0.01)	1.48 (0.01)
Number of chronic conditions	3.69(<0.01)	6.16 (0.01)	6.22(0.01)	7.26 (0.01)
Number of distinct AHRQ	9.74 (<0.01)	19.57 (0.03)	17.48 (0.03)	20.98 (0.02)
HCUP procedure categories^b^				
Total hospital days	0.15 (<0.01)	6.04 (0.05)	3.51 (0.04)	5.62 (0.03)
Distinct providers	8.83 (0.01)	25.34 (0.06)	22.30 (0.06)	27.85 (0.05)
Distinct provider specialties	5.47 (<0.01)	12.43 (0.02)	11.16 (0.02)	13.49 (0.02)
Number of medications in distinct AHFS categories^c^	4.68 (<0.01)	6.20 (0.01)	8.19 (0.01)	7.86 (0.01)

Notes: The differences between the distribution among the spend groups and distribution among the feature levels are all statistically significant (*p* < 0.01). Multinomial chi-square tests were used on categorical features with the null hypothesis of independence between the counts of the spend groups and the counts of the levels. ANOVA tests were used on continuous features with the null hypothesis that the average was the same for all spend groups. We followed the multinomial tests with pairwise tests, chi, and *t*, whose *p*-values we report in the results where appropriate

^a^Socioeconomic status (SES) index is formulated from demographic data on education, housing, income, and employment, mapped to the zip code level per individual. The score is centered at 50.^29^

^b^Agency for Healthcare Research and Quality (AHRQ), Healthcare Cost and Utilization Project (HCUP)^23^.

^c^American Hospital Formulary Service (AHFS)^24^.

^d^Rules used to define persistent individuals prevent any <65 in 2016 from being semi-persistent

The groups show different patterns of healthcare utilization. The semi-persistent group appears to be the most medically complex; this group had the highest number of chronic conditions and frailty (*p*-values <0.001). It also had the greatest overall healthcare utilization (specialists, providers, and procedures from AHRQ\HCUP categories; *p*-values <0.001). The persistent group had the lowest number of hospital days and frailty indicators (*p*-values <0.001) and the highest number of medications from different AHFS categories. Catastrophic individuals had the greatest number of hospital days, followed by semi-persistent individuals (*p*-values <0.001).

**Figure 3:**
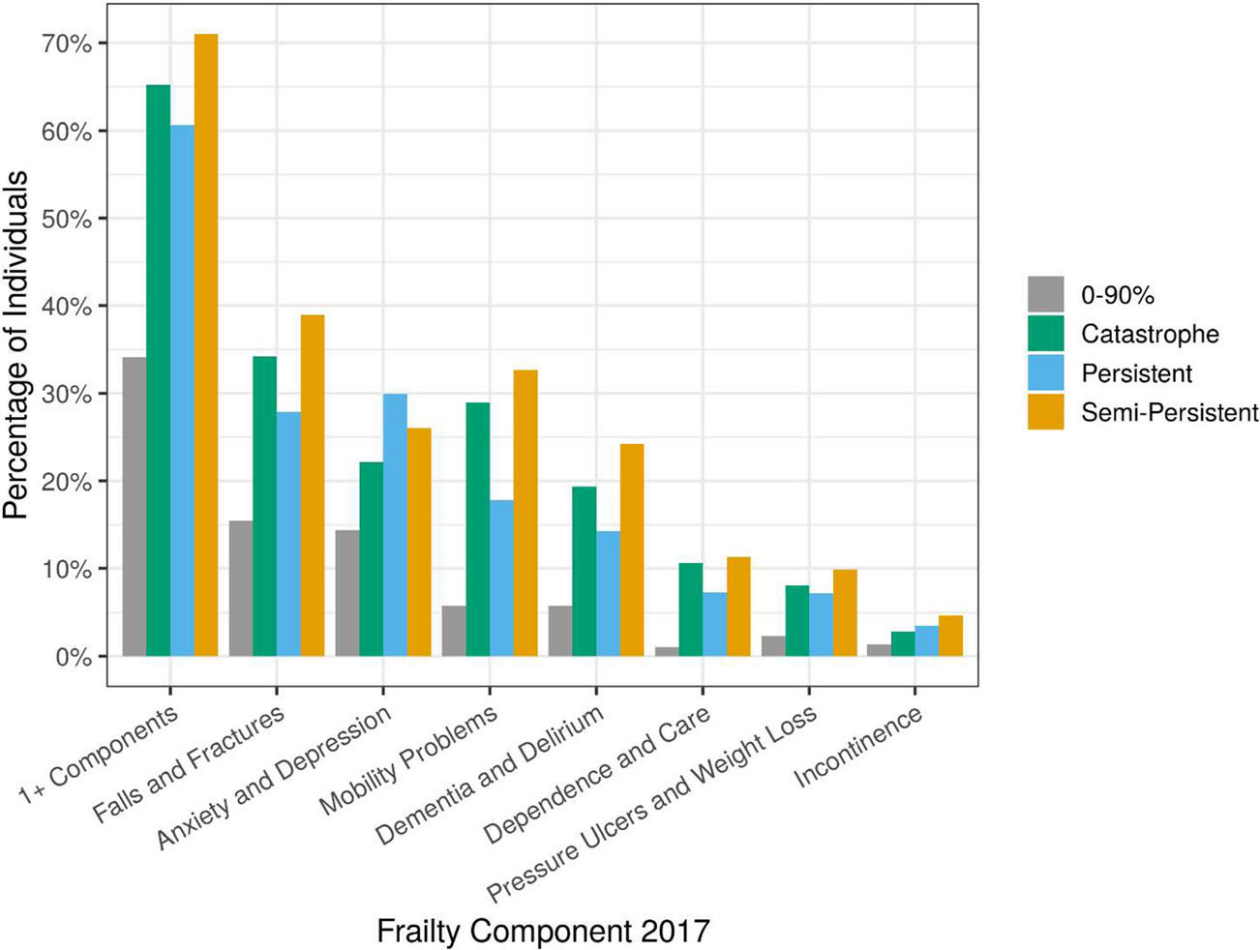
Proportion of individuals with frailty components. Values are shown for 2017, but trends are representative of all years (2017–2019).

Frailty was most prevalent among the semi-persistent group. Roughly 71% of individuals in the semi-persistent group had at least one frailty component (1+ components). The proportion of individuals by frailty component and group in 2017 is shown in Fig. 3. Frailty was also a strong indicator of inpatient cost in the next year for semi-persistent individuals. 2018 semi-persistent enrollees with at least one component of frailty in 2017 and 2018 had inpatient costs 25% higher in 2018 and 34% higher in 2019 on average than those without any components of frailty.

Pharmacy spend was related to persistently high cost; 79% of individuals in the top decile with high proportional pharmacy spend (> 60%) in 2017 remained in the top decile in 2018, and for 79%, their healthcare spend continued to be dominated by pharmacy through 2019. Pharmacy spend was observed to drive a substantial proportion of spend in the high-spend population, accounting for over 60% of total healthcare spend for 18.5% of individuals in the top decile (range 17.5–19.2% in 2017–2019).

**Figure 4:**
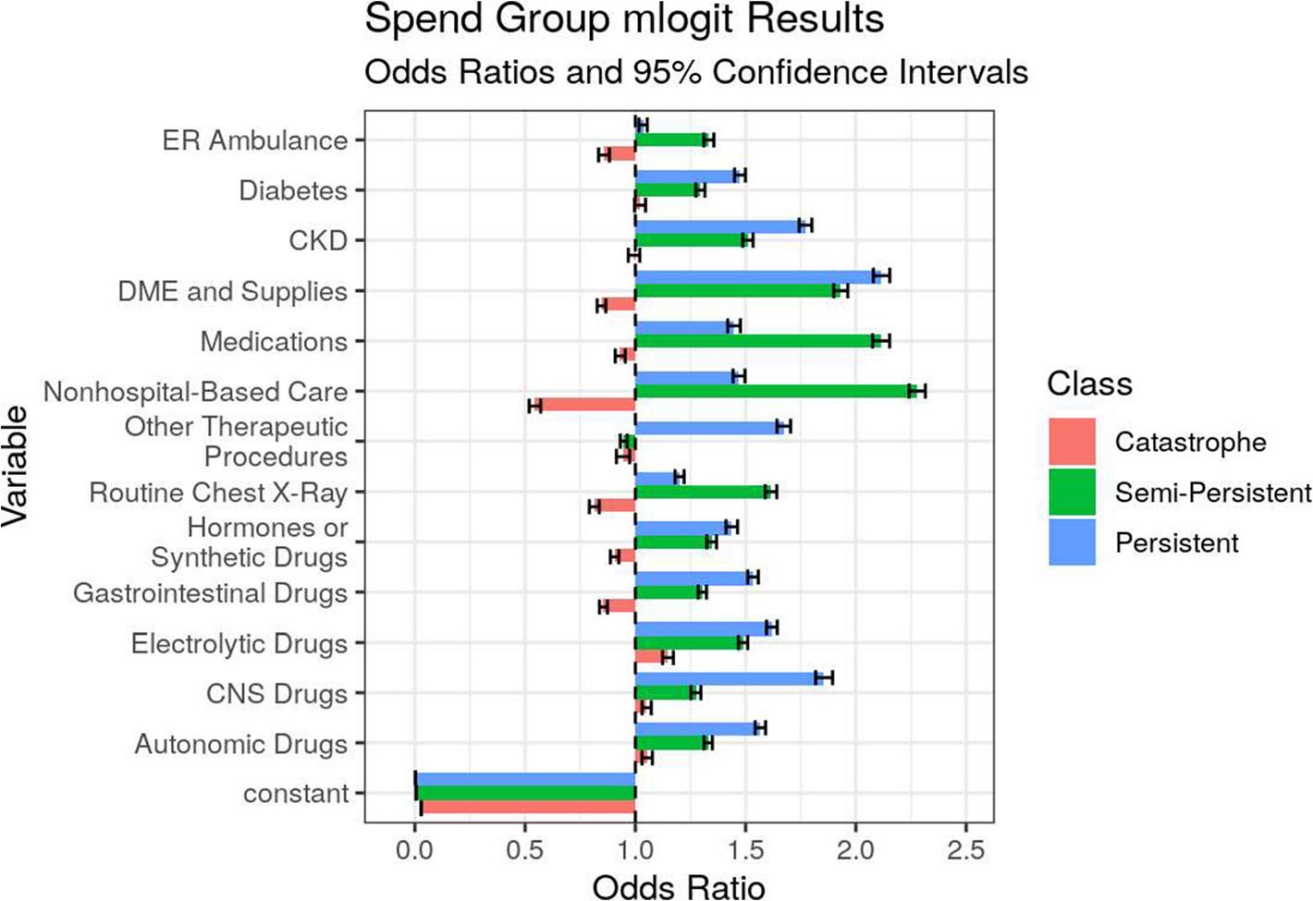
Odds ratios of previous year’s (2016–2018) characteristics tied to current year spend groups for the top decile of spend, 2017–2019. Notes: Features selected by multinomial logistic regression with L1 penalty to minimize collinearity. Odds ratios reflect odds of spend group membership 2017, 2018, and 2019 based on the previous year’s data. More details in Appendix C in the Supplementary information.

The odds ratios in the spend group mlogit model show the effects of the most important characteristics that separate the three groups from the bottom 90% (Fig. 4). Of all features, durable medical equipment (DME) and supplies purchases, CKD, diabetes, and several classes of drug prescriptions give an individual the highest odds of top decile spending and membership in the semi-persistent and persistent classes, relative to the catastrophe and lower spending classes. Non-hospital-based care is the largest anti-indicator of the catastrophe class. It also joins the medication and the chest x-ray procedure classes as the largest indicators of the semi-persistent class over the persistent class.

**Figure 5:**
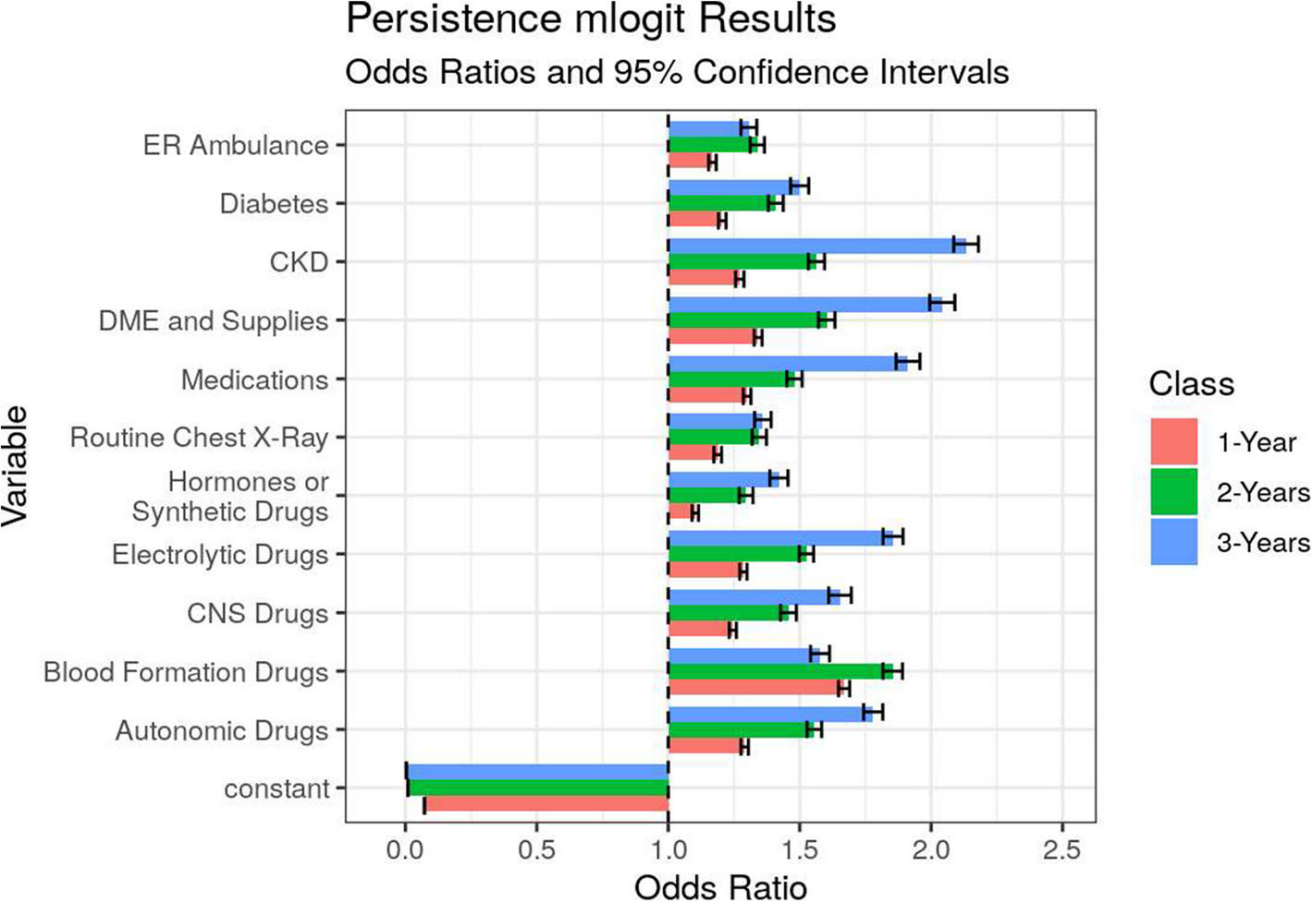
Odds ratios of 2016 characteristics tied to temporal persistence in the top decile of spend 2017–2019. Notes: Features selected by multinomial logistic regression with L1 penalty to minimize collinearity. Odds ratios reflect odds of a certain number of years of spending in the top decile in a three-year period (2017–2019) based on 2016 data. More details in Appendix C in the Supplementary information.

The odds ratios in the mlogit model for persistence similarly show that CKD, diabetes, durable medical equipment purchases, and multiple class of medications are most indicative of individuals with top decile spending in all 3 years, with reference to zero years of top decile spending in 2017–2019 (Fig. 5). Odds ratios and their standard errors and *p*-values are included in Appendix C in the Supplementary information.

In the post hoc analysis that included all deaths, the three groups in the top decile showed different stability and mortality across time (Fig. 2). The catastrophic group comprised 24.6% (*n* = 38,312) of the top decile in 2017 and 80.6% left the top decile the next year, 13.9% dying and the rest splitting evenly between the 0–60th percentile and 60–90th percentile groups. Of those who stayed in the top decile, more individuals moved into the semi-persistent group (12.6%) than the persistent group (6.8%).

The semi-persistent spend group accounted for 42.7% (*n* = 69,431) of individuals in the top decile in 2017. Of these individuals, 68.4% dropped out of the top decile the next year with 18.5% dying and the rest moving mostly into the 60–90th percentiles, 27.8% remained semi-persistent, and 3.7% shifted into the persistent spend group.

The persistent group showed the most stability across time. This group comprised 32.0% (*n* = 54,824) of individuals in the top decile in 2017 and 57.7% remained persistent in 2018 while 12.4% died.

## DISCUSSION

The catastrophic, persistent, and semi-persistent groups show significantly different levels of mortality, frailty, medications, chronic conditions, and healthcare utilization patterns. When examined over 3 years, these groups move in and out of the top decile at different rates. Our findings point to the diversity of the top decile and highlight the implausibility that a singular care delivery model, intervention, or clinical approach will improve care and reduce spending for the top decile.

Semi-persistent member spend is driven by frailty and medical complexity. Frailty poses opportunity for intervention because the physiological, behavioral, or environmental risk factors associated with common frailty components (falls and fractures, mobility problems) are largely modifiable.^30^ Among the semi-persistent group, individuals with greater frailty also had significantly higher hospital spend than non-frail members. These findings contribute to the growing body of research that shows frailty is an important predictor of clinical outcomes, utilization, mortality, and cost.^20,31,32^ Semi-persistent individuals have the most chronic conditions, providers, and specialists. This group is in the top decile based on their medical complexity. We identified 50% of semi-persistent individuals who leave the top decile year to year and do not die. Identifying care patterns and characteristics that are predictive of this movement is an important step in understanding what methods are effective for decreasing costs. The 50% of semi-persistent enrollees who persist in the top decile or die require more targeted interventions or palliative care and reevaluation of contributors to their persistently high cost, especially those that contribute to death.

Catastrophic individuals have low spending in the year prior to entering the top decile. Individuals with acute events (e.g., acute myocardial infarction and hip/pelvic fracture) as well as certain cancers and stroke were more likely to be in this group. The catastrophic group had the most hospital days and few (20–21%) remain in the top decile year to year. There is little opportunity to predict when or why catastrophic individuals will shift into the top decile. Thus, managing high-cost catastrophic patients will require increased patient education and intensive short-term care management.

Most persistent individuals (62–64%) remain in the top decile year to year. The results of the multinomial logistic model on members with the persistent spend support the rules we used to segment this group, which focus on drug usage and ESRD. This stability suggests the need for longitudinal, comprehensive care plans, such as physician-led interdisciplinary teams to provide complex primary and palliative care, as well as home- and community-based services that address behavioral and social determinants of health.^33^ Persistent individuals had significantly more medications than other subgroups; medication management (detecting, resolving, and preventing medication errors and medication-related problems) could be an avenue to reducing cost.^34^

### Limitations

Our findings are conditioned by several limitations. Our analytic approach provides an incomplete picture of an individual’s health because we analyze only medical claims from fee-for-service plans. These data do not provide contextual information such as cognitive abilities, social support needs, and lifestyle characteristics that play a critical role in healthcare costs. Fee-for-service plans provide itemized data for each service which makes for easier analysis but do not represent all Medicare members. Our results are limited by the requirement of continuous enrollment, which biases our sample toward a population with lower mortality and morbidity than the general MA population. We originally excluded the 3–5% of individuals who died annually between 2016 and 2019, a choice informed by other studies.^35^ Including full years of data for individuals dying in later years, the spending threshold of the top decile increases by 15–18%, and ~ 30% of top decile spenders in 2017 die before the end of 2019. We did not consider including partial years, as spending can be time-dependent, given factors such as deductibles and out of pocket maximums, and risks unevenly weighing data. Our data are skewed by a predominantly white sample with a limited representation of other races. In 2019, the racial distribution of the US population aged 65+ was 76% White, 9% Hispanic, 9% Black, 5% Asian, and 1% Native American.^36^ Our sample is also geographically biased because the requirement for fee-for-service and MAPD enrollment inherently over- and underrepresents different states. Future work should prioritize more diverse, representative samples.

## CONCLUSIONS

Our findings are highly applicable to payers, accountable care organizations, and capitated models which have an interest in better understanding and managing the care needs of their most expensive enrollees. To this end, stratification into catastrophe, semi-persistent, and persistent subgroups introduces a useful perspective into how those needs differ.

## Supplementary Information

ESM 1 (PDF 494 kb)
