# Supplementary material for: Temporal Patterns of High-Spend Subgroups Can Inform Service Strategy for Medicare Advantage Enrollees
Source: J Gen Intern Med. 2021 Jun 7;37(8):1853–61. doi: 10.1007/s11606-021-06912-4 (PMC9198168; doi:10.1007/s11606-021-06912-4)
Supplement: Supplementary file 1 — (PDF 494 kb) [file 11606_2021_6912_MOESM1_ESM.pdf]

Appendix A—Database Quality

A.1 Standardization of Data Entry and Data Structure

Medical and pharmacy claims data are captured, predominantly electronically, from sites of care seeking third-party reimbursement for Medicare plans using the industry standard data collection forms HCFA/CMS-1500 for facility claims, UB04/CMS-1450 for professional services and outpatient claims, and NCPDP for pharmacy claims or their electronic equivalents. Structured data from these standardized forms are coded using the International Classification of Diseases, Tenth Revision, Clinical Modification (ICD-10-CM), National Drug Codes (NDC), Current Procedural Terminology (CPT) codes, and Logical Observation Identifiers Names and Codes (LOINC) codes, and Diagnosis Related Groups (DRG). This nomenclature ensures consistency of data collection across geographic regions, health systems, and payers throughout the United States.

A.2 Methods to Control for Errors in Sampling and Data Collection

Claims that do not adhere to the form or coding standards described above are rejected from reimbursement, minimizing the risk that inappropriately structured data are included in the database.

A.3 Data Relevance and Accuracy

Data are transferred into the UnitedHealth Group (UHG) Clinical Discovery Database, where a dedicated team pursues data management to ensure accurate matching of source data to an individual. This protocol uses unique identifiers to match them to existing identifiers in the UHG Clinical Discovery Database to determine whether the individual already exists in the platform. A unique identification number is generated for each individual so that data from multiple

sources can be linked back to that identification number. Individuals that fail to meet the matching criteria are excluded from the UHG Clinical Discovery Database to reduce the risk of erroneous linkage of records. Those whose claims do not fulfill basic standardized data structure requirements described previously are also excluded. During this, all member protected data are stored in a separate database that is only accessible by a designated engineering team. In addition to a persistent identifier being generated for each member, a de-identified primary key is also generated. The de-identified primary key is recycled every six months, at which time each member is assigned a new de-identified primary key. Data that are made available for research through the UHG Clinical Discovery Database use the de-identified primary key as the link across data tables. All protected information has been removed, ensuring any research performed is limited to retrospective analysis of de-identified data and accessed in accordance with Health Insurance Portability and Accountability Act regulations.

A.4 Sufficiency of Basic Data

As described above, individuals lacking enough data to be assigned a unique primary key are excluded from the UHG Clinical Discovery Database, as are patients whose claims did not fulfill basic data structure requirements. In a given month in 2019, the UHG Clinical Discovery Database contained one or more claims from 5 million MA enrollees.

A.5 Adequacy of Possible Derived Data

To reduce the risk of introducing error to standardized, structured claims data, derivation of source data within the UHG Clinical Discovery Database is minimal. The Data Integration team loads, formats, and join the data to appropriate dimension tables. Dimension tables are combined with raw claims information to limit the number of times external tables need to be referenced.

Researchers may request derived fields within data tables prepared specifically for a project. This process is managed by the Data Enrichment team, who creates data dictionaries to accompany derived fields. Tables containing derived data are stored separately from raw source data.

A.6 Design of Computer Editing Methods

Access to modify/edit source data is restricted to a subset of data specialists. Each step in the data flow has a restricted list of individuals able to perform any type of editing to the database, and access level varies by team (Data Integration, Data Enrichment). Researchers using the Clinical Discovery Database may not edit any source data or enrichment data. They are instead given access to “sandbox” locations where they may request editing access for the data tables used in their analyses.

A.7 Data Sharing

The data are proprietary and are not available for public use but can be made available to editors and their approved auditors under a data use agreement to confirm the findings of the current study.

Cost decile movement from 2017 to 2018. Numbers and color both represent the proportion of members from each 2017 cost decile that moved into each 2018 spend decile. Similar trends were observed 2016–17 and 2018–19.

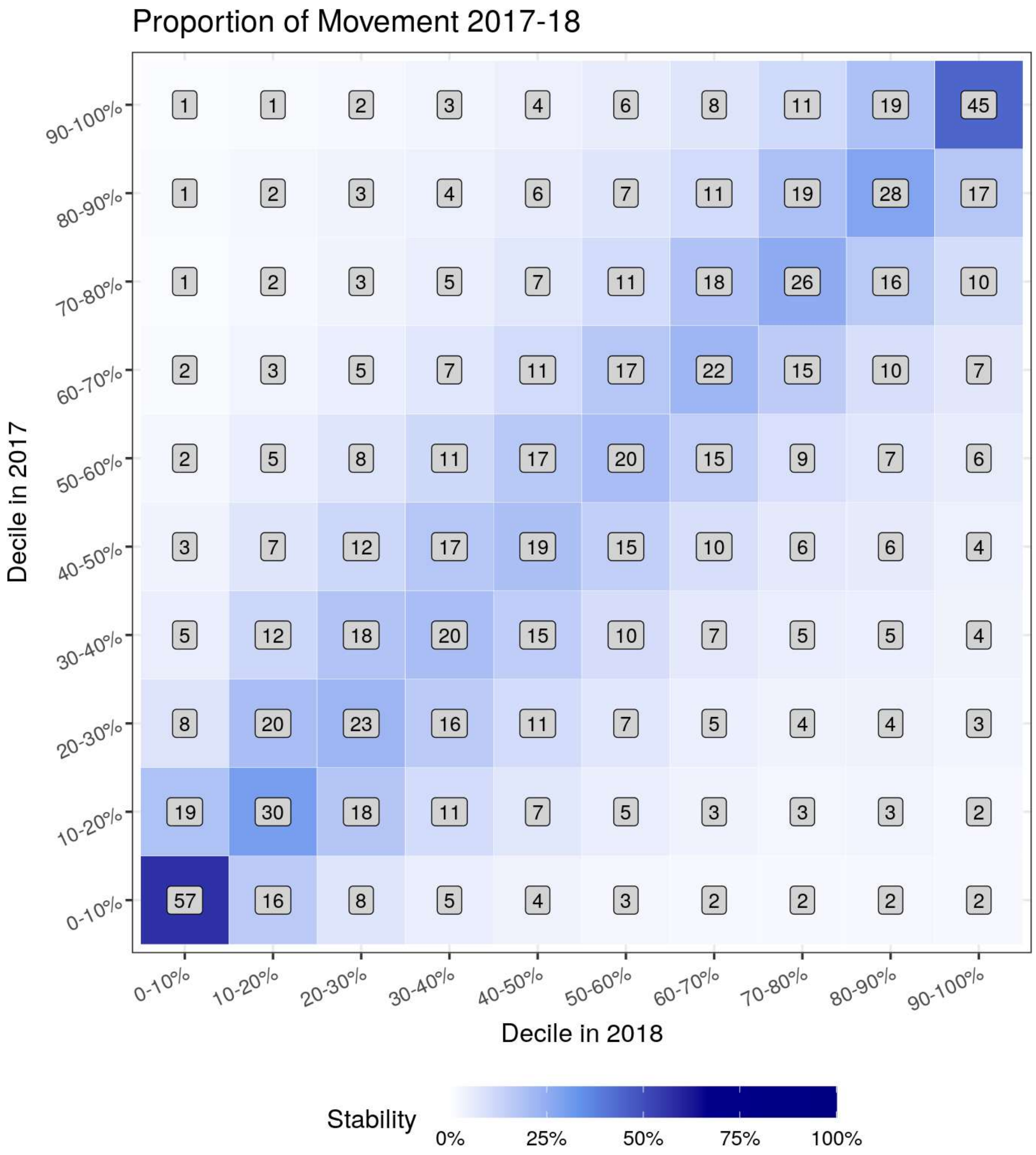

Appendix C—Modeling Methods

B.1 Process Summary

To determine the most important associations between demographic and utilization variables and the temporal persistence of high spend, we run a multinomial logistic regression (mnlogit) on a short list of variables determined previously by a round of feature selection, with top decile spending persistence 2017–2019 (‘0 Years’, ‘1 Year’, ‘2 Years, or ‘3 Years’) as the dependent variable.

B.2 Feature Selection

Feature selection consists of 1000 runs of L1 penalized mnlogits on randomly selected 5% samples of our data. We then take the features that were selected in 950 or more of the runs. The penalty and selection count threshold creates an arbitrary C that selects the 8-12 most influential variables. Python documentation on L1 penalized multinomial logistic regressions here: https://scikit-learn.org/stable/modules/generated/sklearn.linear_model.LogisticRegression.html

B.3 Final Model

The final mnlogit is run with this short list of variables. It is unpenalized and computes odds ratios for each feature and their association with a persistence level relative to ‘0 Years’

Appendix D—Mlogit Results

D.1 Spend Group Model

Below are the log odds results of the multinomial logistic regression computing odds of membership in a class relative to being in the bottom 90% of spend. Variables are lagged one year to the spend group assignment.

	**Catastrophic**		**Persistent**		**Semi-persistent**	
**Variable (Year Prior)**	**Coeff**	**SE**	**Coeff**	**SE**	**Coeff**	**SE**
constant	-3.55	0.01	-5.73	0.01	-5.24	0.01
Autonomic Drugs	0.05	0.01	0.45	0.01	0.29	0.01
CNS Drugs	0.05	0.01	0.62	0.01	0.24	0.01
Electrolytic Drugs	0.14	0.01	0.48	0.01	0.40	0.01
Gastrointestinal Drugs	-0.16	0.01	0.43	0.01	0.27	0.01
Hormones or Synthetic Drugs	-0.10	0.01	0.36	0.01	0.30	0.01
Routine Chest X-Ray	-0.21	0.01	0.18	0.01	0.48	0.01
Other Therapeutic Procedures	-0.06	0.01	0.51	0.01	-0.05	0.01
Nonhospital-Based Care	-0.61	0.01	0.39	0.01	0.82	0.01
Medications	-0.07	0.01	0.37	0.01	0.75	0.01
DME and Supplies	-0.17	0.01	0.75	0.01	0.66	0.01
CKD[a]	-0.01	0.01	0.57	0.01	0.41	0.01
Diabetes[a]	0.02	0.01	0.39	0.01	0.26	0.01
ER Ambulance	-0.15	0.01	0.03	0.01	0.29	0.01

Notes:
[a.] All coefficients are significant to $p < 0.01$, except for coefficients under the Catastrophic class for CKD and Diabetes, which are $p = 0.64$ and $p = 0.09$, respectively.

D.2 Persistence Model

Below are the log odds results of the multinomial logistic regression computing odds of 1, 2, or 3 years of spending in the top 10% relative to no years of spending in the top 10%. Variables are representative of 2016, outcomes are representative of spending 2017–2019.

	**1 Year**		**2 Years**		**3 Years**	
**Variable (2016)**	**Coeff**	**SE**	**Coeff**	**SE**	**Coeff**	**SE**
Constant	-2.63	0.01	-4.62	0.01	-5.66	0.02
Autonomic Drugs	0.26	0.01	0.44	0.01	0.58	0.01
Blood Formation Drugs	0.51	0.01	0.62	0.01	0.46	0.01
CNS Drugs	0.22	0.01	0.38	0.01	0.50	0.01
Electrolytic Drugs	0.25	0.01	0.42	0.01	0.62	0.01
Hormones or Synthetic Drugs	0.10	0.01	0.26	0.01	0.35	0.01
Routine Chest X-Ray	0.17	0.01	0.30	0.01	0.31	0.01
Medications	0.26	0.01	0.39	0.01	0.65	0.01
DME and Supplies	0.29	0.01	0.47	0.01	0.71	0.01
CKD	0.24	0.01	0.45	0.01	0.76	0.01
Diabetes	0.19	0.01	0.34	0.01	0.41	0.01
ER Ambulance	0.16	0.01	0.29	0.01	0.27	0.01

Notes:

- All coefficients are significant to $p < 0.01$.
